# Uptake of the World Health Organization’s trauma care guidelines: a systematic review

**DOI:** 10.2471/BLT.15.162214

**Published:** 2016-05-13

**Authors:** Lacey LaGrone, Kevin Riggle, Manjul Joshipura, Robert Quansah, Teri Reynolds, Kenneth Sherr, Charles Mock

**Affiliations:** a Harborview Injury Prevention and Research Center, Campus Box #356410, University of Washington, Seattle, WA 98104, United States of America (USA).; b Department of Surgery, University of Washington, Seattle, USA.; c Academy of Traumatology, Ahmedabad, India.; d Kwame Nkrumah University of Science and Technology, Kumasi, Ghana.; e World Health Organization, Geneva, Switzerland.; f Department of Global Health, University of Washington, Seattle, USA.

## Abstract

**Objective:**

To understand the degree to which the trauma care guidelines released by the World Health Organization (WHO) between 2004 and 2009 have been used, and to identify priorities for the future implementation and dissemination of such guidelines.

**Methods:**

We conducted a systematic review, across 19 databases, in which the titles of the three sets of guidelines – *Guidelines for essential trauma care*, *Prehospital trauma care systems* and *Guidelines for trauma quality improvement programmes* – were used as the search terms. Results were validated via citation analysis and expert consultation. Two authors independently reviewed each record of the guidelines’ implementation.

**Findings:**

We identified 578 records that provided evidence of dissemination of WHO trauma care guidelines and 101 information sources that together described 140 implementation events. Implementation evidence could be found for 51 countries – 14 (40%) of the 35 low-income countries, 15 (32%) of the 47 lower-middle income, 15 (28%) of the 53 upper-middle-income and 7 (12%) of the 59 high-income. Of the 140 implementations, 63 (45%) could be categorized as needs assessments, 38 (27%) as endorsements by stakeholders, 20 (14%) as incorporations into policy and 19 (14%) as educational interventions.

**Conclusion:**

Although WHO’s trauma care guidelines have been widely implemented, no evidence was identified of their implementation in 143 countries. More serial needs assessments for the ongoing monitoring of capacity for trauma care in health systems and more incorporation of the guidelines into both the formal education of health-care providers and health policy are needed.

## Résumé

**Objectif:**

Comprendre dans quelle mesure ont été utilisées les lignes directrices pour les soins en traumatologie, publiées par l'Organisation mondiale de la Santé (OMS) entre 2004 et 2009, et définir des priorités pour la mise en œuvre et la diffusion futures de ces lignes directrices.

**Méthodes:**

Nous avons procédé à une revue systématique de 19 bases de données, en utilisant les titres anglais des trois séries de lignes directrices – *Guidelines for essential trauma care*, *Prehospital trauma care systems* et *Guidelines for trauma quality improvement programmes* – comme termes de recherche. Les résultats ont été validés par une analyse de citations et une consultation d'experts. Deux auteurs ont revu de façon indépendante chaque occurrence d'application des lignes directrices.

**Résultats:**

Nous avons relevé 578 occurrences qui mettaient en évidence la diffusion des lignes directrices de l'OMS concernant les soins en traumatologie et 101 sources d'information qui décrivaient 140 cas de mise en œuvre de ces lignes directrices. Nous avons trouvé des éléments indiquant leur mise en œuvre dans 51 pays – 14 (40%) des 35 pays à revenu faible, 15 (32%) des 47 pays à revenu intermédiaire - tranche inférieure, 15 (28%) des 53 pays à revenu intermédiaire - tranche supérieure et 7 (12%) des 59 pays à revenu élevé. Sur les 140 cas de mise en œuvre, 63 (45%) relevaient d’évaluations des besoins, 38 (27%) d’approbations des parties prenantes, 20 (14%) d’intégrations à des politiques et 19 (14%) d’interventions pédagogiques.

**Conclusion:**

Bien que les lignes directrices de l'OMS concernant les soins en traumatologie aient été appliquées dans une large mesure, aucun élément n'a permis de montrer leur mise en œuvre dans 143 pays. Il est nécessaire de réaliser davantage d'évaluations des besoins en série pour le suivi continu des capacités en matière de soins en traumatologie dans les systèmes de santé et d'intégrer davantage ces lignes directrices dans l'éducation formelle des professionnels de santé ainsi que dans les politiques de santé.

## Resumen

**Objetivo:**

Comprender hasta qué punto se han utilizado las directrices sobre la atención de traumatismos publicadas por la Organización Mundial de la Salud (OMS) entre 2004 y 2009 e identificar las prioridades para la futura implementación y difusión de dichas directrices.

**Métodos:**

Se llevó a cabo una revisión sistemática, de 19 bases de datos, en la que se utilizaron como términos de búsqueda los títulos de tres conjuntos de directrices: *Guidelines for essential trauma care*, *Prehospital trauma care systems* y *Guidelines for trauma quality improvement programmes*. Se validaron los resultados a través de un análisis de citas y consultas a expertos. De forma independiente, dos autores revisaron todos los informes de la implementación de las directrices.

**Resultados:**

Se identificaron 578 informes que demostraron la difusión de las directrices sobre la atención de traumatismos de la OMS y 101 fuentes de información que describían 140 casos de implementación. Se pudieron encontrar los casos de implementación de 51 países: 14 (40%) de los 35 países de ingresos bajos, 15 (32%) de los 47 países de ingresos medios, 15 (28%) de los 53 países de ingresos medios-altos y 7 (12%) de los 59 países de ingresos altos. De las 140 implementaciones, 63 (45%) podrían categorizarse como evaluaciones de necesidades, 38 (27%) como avales de partes interesadas, 20 (14%) como incorporaciones en la política y 19 (14%) como intervenciones educativas.

**Conclusión:**

A pesar de que las directrices sobre la atención de traumatismos de la OMS se han implementado con cierta amplitud, no se ha demostrado su implementación en los 143 países. Es necesario realizar más evaluaciones de necesidades para evaluación continuada de la capacidad de la atención de traumatismos en los sistemas sanitarios, así como una mayor incorporación de las directrices tanto en la educación formal de los profesionales sanitarios como en las políticas sanitarias.

## ملخص

**الغرض:**

فهم مدى استخدام المبادئ التوجيهية الصادرة عن منظمة الصحة العالمية في الفترة ما بين عامي 2004 و2009 بشأن رعاية المصابين في الحوادث، وتحديد الأولويات لتنفيذ ونشر مثل هذه المبادئ التوجيهية في المستقبل.

**الطريقة:**

قمنا بإجراء مراجعة منهجية عبر 19 قاعدة للبيانات، تم فيها البحث عن عناوين ثلاث مجموعات من المبادئ التوجيهية – المبادئ التوجيهية للرعاية الأساسية للمصابين في الحوادث، وأنظمة الرعاية للمصابين في الحوادث قبل الذهاب إلى المستشفى، والمبادئ التوجيهية لبرامج تحسين نوعية الرعاية للمصابين في الحوادث – واُستخدمت كمصطلحات للبحث. تم توثيق مصداقية النتائج عبر تحليل الاقتباس والتشاور مع الخبراء. وقام اثنان من المؤلفين بمراجعة كل سجل لعملية تنفيذ المبادئ التوجيهية على نحوٍ مستقل.

**النتائج:**

قمنا بتحديد 578 من السجلات التي أثبتت نشر المبادئ التوجيهية الخاصة بمنظمة الصحة العالمية لرعاية المصابين في الحوادث و101 من مصادر المعلومات التي قدمت وصفًا مشتركًا لـ 140 من حالات التنفيذ. يمكن العثور على دليل التنفيذ بالنسبة لـ 51 بلدًا – 14 (‏40%) من بين 35 بلدًا منخفضة الدخل، و15 (32‏%) من بين 47 بلدًا من الشريحة الدنيا من البلدان متوسطة الدخل، و15 (28‏%) من بين 53 بلدًا من الشرائح العليا من بين البلدان متوسطة الدخل، و7 (‏12%) من بين 59 بلدًا مرتفعة الدخل. ومن بين حالات التنفيذ البالغ عددها 140، يمكن تصنيف 63 (45‏%) منها باعتبارها حالات تقييم للاحتياجات، و38 (27‏%) منها باعتبارها مصادقات من الجهات المعنية، و20 (‏14%) باعتبارها عناصر مدمجة في السياسات، و19 (14‏%) باعتبارها تدخلات للتوعية.

**الاستنتاج:**

بالرغم من تطبيق المبادئ التوجيهية الصادرة عن منظمة الصحة العالمية لرعاية المصابين في الحوادث على نطاق واسع، فلم يتم الوقوف على أي دليل لتنفيذها في 143 بلدًا. هناك حاجة إلى القيام بالمزيد من العمليات التسلسلية لتقييم الاحتياجات من أجل المراقبة المستمرة لإمكانية رعاية المصابين في الحوادث في الأنظمة الصحية وتضمين المبادئ التوجيهية بشكل أكبر في كل من التعليم الرسمي للجهات المقدمة للرعاية الصحية وسياسات الصحة. ****

## 摘要

**目的:**

旨在了解世界卫生组织 (WHO) 在 2004 年至 2009 年期间发布的创伤护理指南的使用程度，并且确定未来实施和传播此类指南的重点。

**方法:**

我们对 19 个数据库进行了系统评价，其中三套指南——*《基础创伤护理指南》*、*《住院前创伤护理系统》*和*《创伤质量改善计划指南》*的标题被用作检索词。 通过引文分析和专家评议对结果进行了验证。 两名作者单独审查了每条指南实施记录。

**结果:**

我们明确了 578 份提供 WHO 创伤护理指南传播证据的记录以及共描述了 140 个实施事件的 101 个信息来源。 我们在 51 个国家找到了实施证据，——14 (40%) 份证据来自 35 个低收入国家；15 (32%) 份来自 47 个中低收入国家；15 (28%) 份来自 53 个中高收入国家；7 (12%) 份来自 59 个高收入国家。 在 140 个实施事件中，63 (45%) 个可归类为需求评估，38 (27%) 个属于利益相关者许可，20 (14%) 个已融入政策中，19 (14%) 个属于教育干预。

**结论:**

虽然 WHO 的创伤护理指南已广泛实施，但是没有发现其在全部 143 个国家实施的证据。 仍需要在卫生系统中提供更多的系列需求评估以提升创伤护理的持续监护能力，此外，应该更广泛地将指南融入卫生保健提供者的正式教育和卫生政策中。

## Резюме

**Цель:**

Определить масштабы применения руководящих принципов травматологической помощи, опубликованных Всемирной организацией здравоохранения (ВОЗ) в период между 2004 и 2009 годом, и приоритеты для дальнейшей реализации и распространения таких принципов.

**Методы:**

Авторами был проведен систематический обзор, охватывающий 19 баз данных. В качестве условий поиска использовались названия трех наборов руководящих принципов: Guidelines for essential trauma care (Руководящие принципы основной травматологической помощи), Prehospital trauma care systems (Системы добольничной травматологической помощи) и Guidelines for trauma quality improvement programmes (Руководящие принципы для программ по улучшению качества травматологической помощи). Результаты были подтверждены путем анализа цитирования и консультации со специалистами. Два автора (независимо друг от друга) рассмотрели каждый документ, касающийся реализации руководящих принципов.

**Результаты:**

Были выявлены 578 документов, которые содержат фактические данные, подтверждающие распространение руководящих принципов травматологической помощи, принятых ВОЗ, и 101 источник информации, в которых в общей сложности описывалось 140 случаев реализации. Фактическое подтверждение реализации было обнаружено для 51 страны: 14 (40%) из 35 стран с низким уровнем дохода, 15 (32%) из 47 стран с уровнем дохода ниже среднего, 15 (28%) из 53 стран с уровнем дохода выше среднего и 7 (12%) из 59 стран с высоким уровнем дохода. Из 140 случаев реализации 63 (45%) могли быть отнесены к категории «оценка потребностей», 38 (27%) — к категории «содействие заинтересованных лиц», 20 (14%) — к категории «включения в стратегическое планирование», 19 (14%) — к категории «образовательные мероприятия».

**Вывод:**

Хотя реализация руководящих принципов ВОЗ в области травматологической помощи носит массовый характер, авторам не удалось обнаружить подтверждение их реализации в 143 странах. Требуется увеличить количество периодических оценок потребностей для постоянного отслеживания потенциала в области травматологической помощи в системах здравоохранения, а также масштабы внедрения руководящих принципов в процесс организованного обучения работников системы здравоохранения и политику в области здравоохранения.

## Introduction

As a result of the unsafe conditions and the relatively poor outcomes once someone is injured in low- and middle-income countries, about 90% of the global burden of injury-related mortality and disability is found in low- and middle-income countries.^1^ The likelihood of death after injury is up to sixfold greater in a low- and middle-income country than in a high-income country.^2^ This disparity can be partially attributed to the relatively poor quality of trauma care in low- and middle-income countries – a problem often exacerbated by poor levels of development, organization and planning and a scarcity of programmes for the improvement of trauma care. The development of dedicated systems of trauma care, such as those to be found increasingly often in high-income countries, can improve outcomes after injury.^3^^–^^7^

The World Health Organization (WHO) has made a concerted effort to address geographical inequalities in trauma care, especially via the development of the Essential Trauma Care Project and the publication of three sets of guidelines. These guidelines – entitled *Guidelines for essential trauma care*, *Prehospital trauma care systems*, and *Guidelines for trauma quality improvement programmes* – were published in 2004, 2005 and 2009, respectively, following consultations with dozens of organizations and hundreds of experts.^8^^–^^11^ Together, these guidelines represent the best of the otherwise very limited guidance available to policy-makers and clinicians, in countries at all economic levels, who are seeking ways to strengthen systems for trauma care. Implementation of these guidelines reflects, at least in part, the status of trauma care globally.

For guidelines, publication does not always translate into application or implementation.^12^ Although WHO publishes dozens of sets of guidelines every year,^13^ the dissemination and implementation of any set of WHO guidelines are rarely investigated in detail.^14^^–^^16^ Each of the sets of guidelines on trauma care that WHO published between 2004 and 2009 was mailed to 2000–3000 recipients – including many public libraries and WHO country offices – and several country offices hosted meetings to facilitate dissemination of the guidelines. However, we know very little about the subsequent use of the guidelines and we therefore conducted an Internet-based search for published articles and grey literature on this topic. By so doing, we hoped to identify gaps in use of the guidelines that need to be addressed and obtain a meta-synthesis of experiences with the guidelines that could help promote improvements in trauma care globally. In the broader context, we also sought to expand the knowledge base regarding the dissemination outcomes and implementation strategies for WHO guidelines in general.

## Methods

The registered protocol for this systematic review (PROSPERO: CRD42014010749) was drafted in accordance with *Preferred reporting items for systematic reviews and meta-analyses* (PRISMA) guidelines.^17^ We used the titles of the three sets of WHO guidelines of interest – “*Guidelines for essential trauma care*”, “*Guidelines for trauma quality improvement programmes*” and “*Prehospital trauma care systems*” – as our search terms. Phrase, verbatim or full-text searches were conducted where possible. Searches were restricted only by date, searching only after the date of publication of the guideline used as the search term. Articles published in Arabic, Chinese, English, French, Portuguese, Russian, Spanish or Vietnamese – i.e. the languages into which any of the three sets of the guidelines is known to have been translated – were eligible for inclusion in our review. A comprehensive search of both published and grey literature was conducted within the CINAHL, Cochrane, Embase, Global Health Database, Global Health Library – Regional Indexes, Google, Google Scholar, Grey Literature Report, OAIster, OpenGrey, ProQuest Conference Papers Index, ProQuest Dissertation and Theses, PubMed, SciELO, Scopus, Web of Science, WHO International Clinical Trials Registry Platform Search Portal, WHO LIS and WorldCat databases. We then contacted 20 experts in the field – i.e. the most frequently cited authors in the articles that we considered to be of interest – and asked them to share any information they may have regarding implementation of the guidelines that was unpublished and/or not available online. Finally, we performed citation analysis, using Google Scholar, Scopus and Web of Science, to detect any additional relevant records that had been missed in the initial database searches.

Information sources were included in our review if they included evidence of the dissemination and/or implementation of at least one of the three sets of guidelines. Citation in an article of any information from a set of guidelines – e.g. a statistic found in the guidelines – was considered to be evidence of the dissemination of that set of guidelines. Any reported application of a set of guidelines – e.g. use of the guidelines in needs assessments and/or educational initiatives – was taken as evidence of the implementation of the guidelines. Information sources that only referred to one or more of the sets of guidelines in the form of a link that readers might follow to access or purchase the guidelines were excluded. We included sources regardless of their apparent quality. If two or more information sources described the same implementation event, only one of them was included in our data analysis. The search for relevant information sources was completed at the end of May 2015.

Two authors extracted data. One author performed the initial search, determined the eligibility of information sources for inclusion in the final analysis and determined which eligible sources provided evidence of implementation of the guidelines and which only gave evidence of the guidelines’ dissemination. Sources providing evidence of dissemination were divided into those that advocated use of WHO guidelines and those that that merely made reference to such guidelines. Implementation was separated into four categories: (i) use of the guidelines for needs assessments, by the comparison of existing practices and resources with those recommended in the guidelines; (ii) the endorsement of the guidelines by national professional societies or other formal bodies; (iii) the use of the guidelines in educational interventions; and (iv) the incorporation of components of the guidelines into policy – as indicated by citation of the guidelines in an official regulatory document at an institutional, local or national government level. The same author also categorized each information source that documented implementation of WHO guidelines according to its type. The other author – chosen for his lack of involvement in trauma, quality improvement or WHO and his previous lack of a professional relationship with any of the other authors or advisors – then reviewed the information sources that the first reviewer had classified as defining implementation and independently categorized any implementation. Discordance between the two authors was resolved through discussion – sometimes following referral to a third author. Data were organized using RefWorks reference management software (ProQuest, Ann Arbor, United States of America) and a simple database in Excel (Microsoft, Redmond, USA).

The study was conducted with the assistance of an advisory group that comprised a health-care librarian and five experts in trauma care, trauma quality improvement, WHO guideline formation and dissemination, and systematic review method.

## Results

**Fig. 1:**
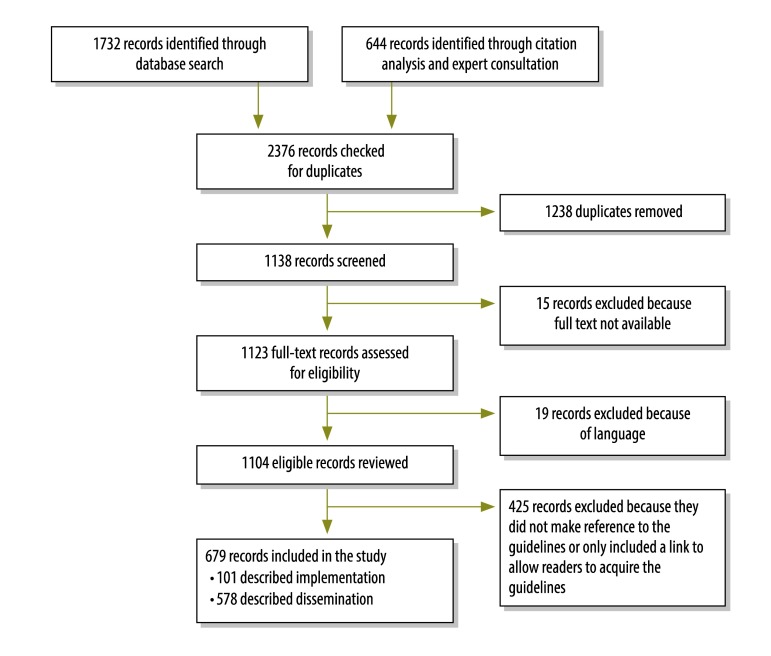
Flow diagram depicting the search results and data extraction of the systematic review on the use the World Health Organization’s trauma care guidelines

Although 2376 records were reviewed for inclusion in the study, only 679 remained after the elimination of duplicates, records without access to full text, texts in excluded languages and records that simply indicated how readers could acquire the guidelines, (Fig. 1). Of the eligible records, 101 (Table 1; available at: http://www.who.int/bulletin/volumes/94/8/15-162214) described 140 unique implementation events whereas the other 578 provided evidence of dissemination of WHO guidelines but not implementation (Table 2). More implementation events for the *Guidelines for essential trauma care* were recorded as needs assessments,^18^^–^^58^^,^^80^^,^^90^ than as stakeholder recommendations^27^^,^^38^^,^^49^^,^^52^^,^^59^^–^^74^^,^^101^ or incorporations into policy^18^^,^^27^^,^^31^^,^^37^^,^^75^^–^^79^^,^^81^^,^^82^ or educational interventions.^37^^,^^40^^,^^53^^,^^63^^,^^83^^–^^89^ Similarly, more implementation events for the *Prehospital trauma care systems* guidelines were recorded as needs assessments,^27^^,^^90^^–^^99^ than as stakeholder endorsements,^60^^,^^64^^,^^81^^,^^100^^–^^104^ or incorporation into policy^27^^,^^90^^,^^105^ or educational interventions.^106^^–^^109^ In contrast, according to our review, *Guidelines for trauma quality improvement programmes* had been implemented mostly as stakeholder endorsements^47^^,^^64^^,^^82^^,^^101^^,^^110^^,^^111^^,^^118^ or in educational interventions^111^^–^^113^^,^^117^ and relatively rarely in needs assessments^35^^,^^47^^,^^52^^,^^114^ or incorporations into policy.^115^^,^^116^ The implementation events and the countries in which they occurred are summarized in Table 3.

**Table 1:** Records reporting on implementation of the World Health Organization’s three sets of trauma care guidelines

Record	Country(ies) or region	Reporting on guideline		
		GETC	GTQIP	PTCS
Gitelman, 2013^18^	Europe	Yes	No	No
Wesson, 2013^19^	Kenya	Yes	No	No
Masella, 2008^20^	Brazil	Yes	No	No
Atiyeh, 2010^21^	LMICs	Yes	No	No
Mock, 2006^22^	Ghana, India, Mexico and Viet Nam	Yes	No	No
Razzak, 2015^23^	Pakistan	Yes	No	No
Son, 2007^24^	Viet Nam	Yes	No	No
Rosales-Mayor, 2011^25^	Peru	Yes	No	No
Chichom-Mefire, 2014^26^	Cameroon	Yes	No	No
Mock, 2009^27^	Colombia, Ecuador, India, Latin America and Mozambique	Yes	No	Yes
Hsiao, 2013^28^	India	Yes	No	No
Tachfouti, 2011^29^	Morocco	Yes	No	No
Remick, 2014^30^	South Sudan	Yes	No	No
Hardcastle, 2013^31^	South Africa	Yes	No	No
Parra, 2013^32^	Latin America	Yes	No	No
Sawaya, 2013^33^	Lebanon	Yes	No	No
Aboutanos, 2012^34^	Ecuador	Yes	No	No
O’Reilly, 2013^35^	Armenia, Cambodia, China, Croatia, Ethiopia, Ghana, Haiti, India, Iran (Islamic Republic of), Jamaica, Kenya, Malawi, Malaysia, Mexico, Nicaragua, Nigeria, Pakistan, Saudi Arabia, South Africa, Thailand and Uganda	Yes	No	No
Baker, 2013^36^	United Republic of Tanzania	Yes	No	No
Son, 2006^37^	Viet Nam	Yes	No	No
Goosen, 2006^38^	Mozambique	Yes	No	No
Nakahara, 2009^39^	Cambodia	Yes	No	No
Pringle, 2012^40^	Nicaragua	Yes	No	No
Arreola-Risa, 2006^41^	Mexico	Yes	No	No
Hanche-Olsen, 2012^42^	Botswana	Yes	No	No
Notrica, 2011^43^	Rwanda	Yes	No	No
Essential Trauma Care Project, 2014^44^	Global	Yes	No	No
Asheel, 2010^45^	India	Yes	No	No
Hanche-Olsen, 2015^46^	Botswana	Yes	No	No
Hardcastle, 2014^47^	Botswana	Yes	No	Yes
Quansah, 2004^48^	Ghana	Yes	No	No
Joshipura, 2006^49^	India	Yes	No	No
Nouh, 2014^50^	Kuwait	Yes	No	No
Zwi, 2008^51^	Timor-Leste	Yes	No	No
Clarke, 2014^52^	South Africa	Yes	No	Yes
Jayaraman, 2009^53^	Uganda	Yes	No	No
Okada, 2010^54^	Viet Nam	Yes	No	No
Shah, 2015^55^	India	Yes	No	No
Burke, 2014^56^	Kenya	Yes	No	No
Ogunniyi, 2015^57^	South Sudan	Yes	No	No
Ankomah, 2015^58^	Ghana	Yes	No	No
Neira, 2011^59^	Argentina	Yes	No	No
Mould-Millman, 2014^60^	Africa	Yes	Yes	No
Mock, 2006^61^	Mexico and Sri Lanka	Yes	No	No
Bellagio, 2008^62^	Uganda	Yes	No	No
Advanced Trauma Training Program, 2014^63^	Nigeria	Yes	No	No
Widmer, 2014^64^	Global	Yes	Yes	Yes
WHO, 2011^65^	Global	Yes	No	No
American Society of Health-System Pharmacists, 2014^66^	United States of America	Yes	No	No
Gitelman, 2008^67^	Europe	Yes	No	No
Potokar, 2013^68^	LMICs	Yes	No	No
Sethi, 2006^69^	Europe	Yes	No	No
WHO, 2004^70^	Africa	Yes	No	No
Syracuse University, 2016^71^	India	Yes	No	No
Quansah, 2006^72^	Ghana	Yes	No	No
WHO, 2008^73^	Global	Yes	No	No
WHO, 2008^74^	Global	Yes	No	No
International Campaign to Ban Landmines, 2005^75^	Mozambique	Yes	No	No
Villanueva, 2010^76^	Mexico	Yes	No	No
Thota, 2005^77^	India	Yes	No	No
O’Reilly, 2008^78^	Sri Lanka	Yes	No	No
Mock, 2011^79^	Cambodia, Ecuador, Ghana and Sri Lanka	Yes	No	No
Stewart, 2014^80^	Ghana	Yes	No	No
WHO, 2010^81^	Africa	Yes	Yes	No
Ministry of Health Lisbon, 2003^82^	Portugal	Yes	No	Yes
Charlton, 2011^83^	Sri Lanka	Yes	No	No
Tchorz, 2007^84^	India	Yes	No	No
University of Ibadan, 2014^85^	Nigeria	Yes	No	No
Foletti, 2014^86^	Burkina Faso, Senegal and Sierra Leone	Yes	No	No
Chinese Nursing, 2007^87^	China	Yes	No	No
Liberia Emergency Medicine Elective, 2014^88^	Liberia	Yes	No	No
O’Reilly, 2011^89^	India and Sri Lanka	Yes	No	No
Aboutanos, 2010^90^	Ecuador	Yes	Yes	No
Goniewicz, 2011^91^	Poland	No	Yes	No
Mould-Millman, 2011^92^	Ghana	No	Yes	No
Adeloye, 2012^93^	Nigeria	No	Yes	No
Nielsen, 2012^94^	Brazil, Colombia, Ecuador, Ghana, India, Kenya, Mexico, Pakistan, Panama, Peru, South Africa, Sri Lanka and Viet Nam	No	Yes	No
Risiva, 2009^95^	South Africa	No	Yes	No
Baqir, 2011^96^	Pakistan	No	Yes	No
Ismail, 2012^97^	Malaysia	No	Yes	No
Bhatti, 2013^98^	Pakistan	No	Yes	No
Challoner, 2013^99^	Liberia	No	Yes	No
Panamerican Trauma Society, 2014^100^	Americas	No	Yes	No
Mahendra, 2012^101^	Global	Yes	Yes	Yes
Gururaj, 2014^102^	India	No	Yes	No
Hardcastle, 2011^103^	South Africa	No	Yes	No
Friesen, 2011^104^	LMICs	No	Yes	No
French Senate, 2015^105^	France	No	Yes	No
Jayaraman, 2009^106^	Uganda	No	Yes	No
Schuetz, 2014^107^	Bolivia (Plurinational State of)	No	Yes	No
El Sayed, 2013^108^	Lebanon	No	Yes	No
Geduld, 2011^109^	Madagascar	No	Yes	No
Neurotrauma Society of India, 2010^110^	India	No	No	Yes
Åkerström, 2012^111^	Global and Kenya	No	No	Yes
O’Reilly, 2013^112^	Myanmar	No	No	Yes
Panamerican Trauma Society, 2012^113^	Americas	No	No	Yes
Schoeneberg, 2014^114^	Germany	No	No	Yes
Yeboah, 2014^115^	Ghana	No	No	Yes
Tozija, 2013^116^	The former Yugoslav Republic of Macedonia	No	No	Yes
O’Reilly, 2014^117^	Sri Lanka	No	No	Yes
Oakley, 2015^118^	United Kingdom	No	No	Yes

GETC: *Guidelines for essential trauma care*; GTQIP: *Guidelines for trauma quality improvement programmes*; LMIC: low- and middle-income countries; PTCS: *Prehospital trauma care systems*; WHO: World Health Organization.

**Table 2:** Implementation and dissemination of the World Health Organization’s three sets of trauma care guidelines

Event^a^	No. (%)			
	GETC	GTQIP	PTCS	Total
**Implementation**				
All types	94 (100)	17 (100)	29 (100)	140 (100)
Needs assessments	45 (48)	5 (29)	13 (45)	63 (45)
Stakeholder endorsements	24 (26)	6 (35)	8 (28)	38 (27)
Educational interventions	11 (12)	4 (24)	4 (14)	19 (14)
Policy developments	14 (15)	2 (12)	4 (14)	20 (14)
**Dissemination**				
All types	346 (100)	56 (100)	176 (100)	578 (100)
With advocacy	58 (17)	10 (18)	22 (12)	90 (16)
With guidelines only referenced	288 (83)	46 (82)	154 (88)	488 (84)

GETC: *Guidelines for essential trauma care*; GTQIP: *Guidelines for trauma quality improvement programmes*; PTCS: *Prehospital trauma care systems*.

^a^ Each event was traced during a systematic review, of published and grey literature, that covered the period from the release of the first set of guidelines – i.e. the *Guidelines for essential trauma care*, which were published in 2004 – to the end of May 2015.

**Table 3:** Examples of the implementation of the World Health Organization’s trauma care guidelines

Income group, country or region	Reported implementation events
**Low-income**	
Burkina Faso	GETC incorporated into an educational module for humanitarian aid workers.^86^
Cambodia	GETC used to develop questionnaires that were administered in a nationally representative sample of 85 health centres and 17 referral hospitals.^39^ The same guidelines were used by ministry of health planners.^79^ Published reports of trauma registries were evaluated using a tool derived from GETC and GTQIP.^35^
Ethiopia	Published reports of trauma registries were evaluated using a tool derived from GETC and GTQIP.^35^
Haiti	Published reports of trauma registries were evaluated using a tool derived from GETC and GTQIP.^35^
Liberia	GETC were required reading for the resident physicians taking an online course in emergency medicine.^88^ An assessment of an emergency department in Monrovia was compared with the standards defined in PTCS guidelines.^99^ GETC and/or GTQIP used to conduct one-day courses for trauma care providers.^111^
Madagascar	A course based on PTCS guidelines was taught to taxi drivers, as part of a plan to develop a system of lay first-responders.^109^
Malawi	Published reports of trauma registries were evaluated using a tool derived from GETC and GTQIP.^35^
Mozambique	WHO, national and local government and other external expert representatives conducted a case review, of the trauma system in Maputo, that was based on criteria from GETC and PTCS guidelines. The results led to recommendations for strengthening the trauma system – including injury surveillance.^27^^,^^38^^,^^75^
Rwanda	GETC used to develop a survey tool to assess the surgical and anaesthesia infrastructure at 21 district-level hospitals.^43^
Sierra Leone	GETC incorporated into an educational module for humanitarian aid workers.^86^
Uganda	GETC and PTCS guidelines incorporated into survey of providers of prehospital care in Kampala and subsequently used as the foundations of a lay first-responders’ course.^53^^,^^106^ A professional society report – from the Bellagio Essential Surgery Group – committed to the revision and adaption of GETC and PTCS guidelines.^62^ Published reports of trauma registries were evaluated using a tool derived from GETC and GTQIP.^35^
United Republic of Tanzania	GETC used in the formation of a survey tool used to assess ten hospitals.^36^ GETC and GTQIP used, by a PhD student in an ongoing project, to investigate the suitability of local trauma system development.^47^
**Lower-middle-income**	
Armenia	Published reports of trauma registries were evaluated using a tool derived from GETC and GTQIP.^35^
Bolivia (Plurinational State of)	PTCS guidelines used as the basis for a lay first-responders’ course.^107^
Cameroon	GETC used to create a tool to assess the physical and human resources and organizational capacity of district hospitals in the Central region.^26^
Ghana	GETC used by ministry of health planners^27^ and served as the basis for a high-profile stakeholders meeting that resulted in a set of policy recommendations that were presented to parliament.^72^ The same guidelines used to assess physical resources for trauma care,^48^^,^^80^ including, specifically, for paediatric trauma care.^58^ Published reports of trauma registries were evaluated using a tool derived from GETC and GTQIP.^35^ PTCS guidelines adapted to test the knowledge of emergency medical technicians in Accra^92^ and served as the basis for a survey, on the status of prehospital care, that was distributed to the leaders of emergency medical services.^94^ GTQIP implemented, via the institution of preventable death panels, at an academic hospital.^115^
India	GETC used for needs assessments of trauma care capabilities nationally,^22^^,^^49^ targeted in Alappuzha district^45^ or with a focus on either human resources^28^ or the availability of technology.^55^ In 2003, in Gujarat, the department of health, a WHO subcountry office and representatives of local and international professional groups held a meeting to adapt GETC to local circumstances.^77^ A similar meeting regarding implementation strategies was held in 2005.^27^ GETC were endorsed by the Academy of Traumatology^27^ and referenced in a working paper, commissioned by the government, that made recommendations for stabilizing the trauma system.^71^ GETC used to assess a training programme for trauma teams^89^ and incorporated into a pilot two-day intensive trauma course for physicians in Bangalore.^84^ Published reports of trauma registries were evaluated using a tool derived from GETC and GTQIP.^35^ PTCS guidelines served as basis for a survey, on the status of prehospital care, that was distributed to the leaders of emergency medical services.^94^ The same guidelines were referenced in a National Institute of Mental Health and Neurosciences public health alert that recommended development of a first-tier trauma response.^102^ The Secretary of the Neurotrauma Society cited GTQIP in a newsletter article that made an explicit call for increased quality improvement activities.^110^
Indonesia	GETC used to assess the hospital capacities for trauma care in East Timor.^51^
Kenya	GETC used as basis for needs assessment of district and provincial hospitals and health centres^19^^,^^56^ and taught as part of a two-day course for medical providers.^111^ Published reports of trauma registries were evaluated using a tool derived from GETC and GTQIP.^35^ PTCS guidelines served as basis for a survey, on the status of prehospital care, that was distributed to the leaders of emergency medical services.^94^
Morocco	GETC used as the basis for an assessment of a university hospital and its associated prehospital system.^29^
Myanmar	Course materials regarding morbidity and mortality conferences – which were developed from GTQIP – were incorporated into a training course for trauma teams.^112^
Nicaragua	Grant proposal included a needs assessment and the development of an emergency medicine handbook that were based on GETC.^40^ Published reports of trauma registries were evaluated using a tool derived from GETC and GTQIP.^35^
Nigeria	GETC incorporated into an online university curriculum^85^ and recommended for implementation – and cited as a stimulus for external rotations for medical providers – in a programme of training in advanced trauma care.^63^ Published reports of trauma registries were evaluated using a tool derived from GETC and GTQIP.^35^ A conceptual framework for a literature review of the trauma system was based on PTCS guidelines.^93^
Pakistan	GETC and PTCS guidelines used to develop a questionnaire administered to 141 staff members at ambulance stations along an interurban road.^23^^,^^98^ Published reports of trauma registries were evaluated using a tool derived from GETC and GTQIP.^35^ PTCS guidelines were used as standard of comparison for a prehospital system in Karachi^96^ and served as basis for a survey, on the status of prehospital care, that was distributed to the leaders of emergency medical services.^94^
Senegal	GETC incorporated into an educational module for humanitarian aid workers.^86^
Sri Lanka	GETC used by ministry of health planners,^27^ used as a standard in the Health for the South capacity building project,^78^ adapted by the College of Surgeons of Sri Lanka, Sri Lanka Medical Association and the WHO country office^61^ and incorporated into an educational programme for emergency nurses.^83^ PTCS guidelines served as basis for a survey, on the status of prehospital care, that was distributed to the leaders of emergency medical services.^94^ GTQIP were taught, as a one-day course, to health-care providers in Galle.^117^
Sudan	GETC used to evaluate the quality of trauma education for community health workers^57^ and incorporated into a novel Global Trauma Systems Evaluations Tool that was used to identify areas for urgent improvement in a military trauma system.^30^
Viet Nam	GETC used for needs assessments at national, district and provincial hospitals.^22^^,^^24^^,^^37^^,^^54^ The documented response by the health department, to the deficiencies identified, included trauma training programmes for physicians and nurses based on GETC.^37^
**Upper-middle-income**	
Botswana	GETC used as tool, in the 27 government hospitals, to investigate trauma care organization, capacity and quality improvement and the physical resources for trauma care.^42^^,^^46^ GETC and GTQIP used, by a PhD student in ongoing project, to investigate the suitability of local trauma system development.^47^
Brazil	GETC used to assess physical and human resources for care at a regional trauma centre.^20^ PTCS guidelines served as the basis for a survey, on the status of prehospital care, that was distributed to the leaders of emergency medical services.^94^ A continuing education course for health-care professionals was based on GTQIP.^111^
China	GETC were required reading for nursing students enrolled in an online summer elective course.^87^ Published reports of trauma registries were evaluated using a tool derived from GETC and GTQIP.^35^
Colombia	PTCS guidelines used for a needs assessment and subsequently incorporated into national legislation that stipulated basic qualifications for providers, included equipment lists and made audits mandatory.^27^^,^^90^ GETC also used as the basis for a needs assessment.^27^
Ecuador	GETC used in needs assessments, for the general care of trauma and for the care of traumatic brain injury, at 24 sites in seven provinces.^27^^,^^34^ The same guidelines were also endorsed by the Ecuadorian Trauma Society and used by ministry of health planners.^27^^,^^79^ PTCS guidelines served as the basis for a survey, on the status of prehospital care, that was distributed to the leaders of emergency medical services.^94^
Iran (Islamic Republic of)	Published reports of trauma registries were evaluated using a tool derived from GETC and GTQIP.^35^
Jamaica	Published reports of trauma registries were evaluated using a tool derived from GETC and GTQIP.^35^
Lebanon	GETC used as the basis for a national survey of the resources available for paediatric trauma care.^33^ A plan to train official ministry of health emergency responders to a level defined in PTCS guidelines is being implemented.^108^
Malaysia	Published reports of trauma registries were evaluated using a tool derived from GETC and GTQIP.^35^ The advanced life support equipment available on 1075 ambulances was compared with recommendations in PTCS guidelines.^97^ A continuing education course for health-care professionals was based on GTQIP.^111^
Mexico	GETC used for needs assessments at 16 facilities,^41^ endorsed by the Mexican Association for the Medicine and Surgery of Trauma,^61^ used by ministry of health planners^27^ and referenced in national standards.^76^ Published reports of trauma registries were evaluated using a tool derived from GETC and GTQIP.^35^ PTCS guidelines served as the basis for a survey, on the status of prehospital care, that was distributed to the leaders of emergency medical services^94^ and were subsequently incorporated into national legislation that stipulated basic qualifications for providers, included equipment lists and made audits mandatory.^27^^,^^90^
Panama	PTCS guidelines served as the basis for a survey, on the status of prehospital care, that was distributed to the leaders of emergency medical services.^94^
Paraguay	A continuing education course for health-care professionals was based on GTQIP.^111^
Peru	A semi-structured questionnaire based on GETC was administered to emergency department heads at eight hospitals in Ayacucho, Lima and Pucallpa.^25^ PTCS guidelines served as the basis for a survey, on the status of prehospital care, that was distributed to the leaders of emergency medical services.^94^
South Africa	GETC used as the standard against which the inpatient trauma care facilities in KwaZulu-Natal were compared; the results led to a proposal for the development of a local trauma system.^31^ After GETC and GTQIP were used to assess the resources for trauma care in a rural health district, the Trauma Society of South Africa used the results to recommend the development of trauma registries and improvements in trauma care to the government.^52^ Published reports of trauma registries were evaluated using a tool derived from GETC and GTQIP.^35^ PTCS guidelines served as the basis for a survey, on the status of prehospital care, that was distributed to the leaders of emergency medical services.^94^ They also formed the basis of a separate targeted questionnaire used in Limpopo province,^95^ and recommendations on national guidelines for assessment of trauma centres.^103^
Thailand	Published reports of trauma registries were evaluated using a tool derived from GETC and GTQIP.^35^ A continuing education course for health-care professionals was based on GTQIP.^111^
The former Yugoslav Republic of Macedonia	The findings of a needs assessment based on the GTQIP were integrated into official strategy for emergency medical services 2009–2017.^116^
**High-income**	
Argentina	GETC formed the foundations of a 2010 consensus statement by the Intersociety Coalition for the Professional Certification, Categorization and Institutional Accreditation in Trauma, Emergency and Disasters.^59^
Croatia	Published reports of trauma registries were evaluated using a tool derived from GETC and GTQIP.^35^
France	PTCS guidelines referenced in national legislation, proposed in 2009, that was designed to add basic training in first aid to the requirements for acquiring a driver’s licence.^105^
Germany	The definition of preventable from GTQIP was used in a study of mortality among injured children in a trauma centre.^114^
Poland	PTCS guidelines used, for comparison, in an assessment of the adequacy of the injury response system.^91^
Portugal	GETC and GTQIP referenced seven times and twice, respectively, in national norms.^82^
Saudi Arabia	Published reports of trauma registries were evaluated using a tool derived from GETC and GTQIP.^35^ GETC also used to assess trauma care services in the capital, Riyadh.^50^
United Kingdom	GTQIP referenced in the Royal College of Anaesthetists’ professional guidelines that recommended preventable death panels, governance meetings and morbidity and mortality meetings.^118^
United States of America	American Society of Health-System Pharmacists recommends use of GETC.^66^
**Region**	
Global	Geneva declaration policy paper recommends GTQIP implementation.^64^ National Center for Injury Prevention and Control works with national and international public health partners to promote GTQIP implementation.^101^ WHO published GETC as checklist to facilitate use as needs assessment.^44^ WHO/Global Health Workforce Alliance/UNICEF/IFRC/ UNHRC recommend use of GETC in joint statement regarding scale-up of community-based health workforce.^65^ GETC recommended in WHO’s *Speed Management: A Road Safety Manual for Decision-Makers and Practitioners*.^73^ GETC recommended in WHO’s *Preventing violence and reducing its impact.*^74^
Africa	African Federation for Emergency Medicine recommended implementation of GETC and PTCS in workgroup consensus paper.^60^ Executive board report of the WHO regional director describes plans to implement GETC and PTCS at regional and country level.^70^^,^^81^
Americas	Panamerican Trauma Society hosts course based on GTQIP accessible to providers throughout the Americas.^113^ GETC used in survey of trauma care resources in Latin America.^32^ PTCS serves as “basis of efforts” of Panamerican Trauma Society Pre-hospital sub-committee.^100^
Europe	The European Union SafetyNet project developed and recommended the use of road safety performance indicators based on the GETC.^18^^,^^67^ WHO regional office white paper on Injuries and Violence in Europe makes recommendations based on GETC.^69^
**Income group**	
LMICs	GETC used as reference for review of access to essential surgical services in LMICs.^21^ International Network for Training Education and Reseach in Burns used GETC as framework for development of 2013 standards for burn care services.^68^ Trek Medics, an international NGO, recommends use of PTCS.^104^

GETC: *Guidelines for essential trauma care*; GTQIP: *Guidelines for trauma quality improvement programmes*; IFRC: International Federation of Red Cross; LMICs: low and middle-income countries; NGO: nongovernmental organization; PTCS: *Prehospital trauma care systems*; UNICEF: United Nations Children’s Fund; UNHRC: United Nations Human Rights Council; WHO: World Health Organization.

Of the 19 descriptions of inclusion of the guidelines in the curriculum of an educational intervention, nine described continuing medical education for professionals,^37^^,^^63^^,^^83^^–^^85^^,^^108^^,^^111^^,^^113^^,^^117^ four described courses for lay first-responders,^53^^,^^107^^,^^109^ another four described education of postgraduate physicians in training,^40^^,^^86^^,^^88^^,^^112^ and one the education of nursing students.^87^ One reference described use of the guidelines to audit existing educational practices.^89^ Only one of the educational interventions described inclusion of the WHO guidelines in degree requirements.^112^

Approximately half of the eligible information sources were journal articles listed by PubMed and most of the remainder were from grey literature (Table 4). Our analysis also included 13 implementation events that were only reported directly to us, by the 20 experts in the field who we contacted.^46^^,^^47^^,^^50^^,^^52^^,^^59^^,^^112^^–^^117^

**Table 4:** Sources of information on the implementation of the World Health Organization’s three sets of trauma care guidelines, included in the systematic review

Source type	No. of implementation events (%)			
	GETC	GTQIP	PTCS	All guidelines
Journal covered by PubMed	54 (57)	5 (29)	18 (62)	77 (55)
Other journal	5 (5)	0 (0)	2 (7)	7 (5)
Professional society report	2 (2)	5 (29)	2 (7)	9 (6)
Web page or blog	5 (5)	0 (0)	2 (7)	7 (5)
Conference proceedings	2 (2)	1 (6)	0 (0)	3 (2)
Thesis	1 (1)	0 (0)	2 (7)	3 (2)
WHO report	7 (8)	0 (0)	1 (3)	8 (6)
Government report	2 (2)	0 (0)	1 (3)	3 (2)
Curriculum	3 (3)	0 (0)	0 (0)	3 (2)
Grant	2 (2)	0 (0)	0 (0)	2 (1)
National policy	2 (2)	0 (0)	0 (0)	2 (1)
Report	2 (2)	0 (0)	1 (3)	3 (2)
Expert consultation	7 (8)	6 (35)	0 (0)	13 (9)
**Total**	**94 (100)**	**17 (100)**	**29 (100)**	**140 (100)**

GETC: *Guidelines for essential trauma care*; GTQIP: *Guidelines for trauma quality improvement programmes*; PTCS: *Prehospital trauma care systems*; WHO: World Health Organization.

**Fig. 2:**
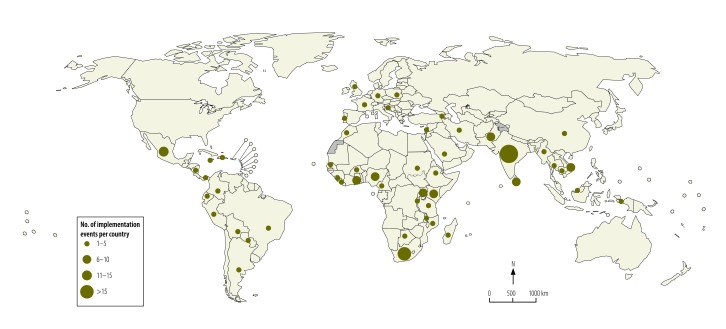
Geographical distribution of the implementation events for the World Health Organization’s three sets of trauma care guidelines, as traced in the systematic review

Notes: The filled circles indicate the number of implementation events recorded in each country and not the exact locations of those events within each country. The three sets of trauma guidelines are: *Guidelines for essential trauma care*, *Guidelines for trauma quality improvement programmes* and *Prehospital trauma care systems*.

According to our analysis, at least one of the three sets of guidelines we investigated had been implemented in each of at least 51 countries – with evidence of implementation in 14 (40%) of the 35 low-income countries, 15 (32%) of the 47 lower-middle income, 15 (28%) of the 53 upper-middle-income and 7 (12%) of the 59 high-income. The location of several implementation events could only be identified as low- and middle-income countries,^21^^,^^68^^,^^104^ Africa,^60^^,^^70^^,^^81^ Europe,^18^^,^^67^^,^^69^ Latin America^27^^,^^32^ or, even more broadly, the Americas.^100^^,^^113^ The number of implementation events recorded per country varied, with more than 10 such events reported in each of five countries: Ghana, India, Mexico, South Africa and Viet Nam (Fig. 2).

Almost all (134; 96%) of the 140 implementation events we included in our analysis had been reported in English. Of the 33 reports of implementation events in Latin America that we included, only three were in Spanish and only one was in Portuguese. Similarly, only two of the 16 reports of implementation events in Francophone countries that we included were in French.

Although the three sets of guidelines were specifically developed for low- and middle-income countries, at least one of the sets had been implemented in each of several high-income countries. In France, for example, the senate had adopted a draft bill to include training in first aid in the requirements for a driver’s licence and that bill had made reference to *Prehospital trauma care systems*.^105^

## Discussion

When we planned this systematic review, our main aim was to determine the extent to which the WHO guidelines on trauma care were being used. The results of the review indicate fairly widespread implementation of the guidelines, with implementation events of various types documented in 51 countries – including 40% of all low-income countries and 30% of all middle-income countries. However, only a small portion (14%) of the relevant implementation events that we did trace involved the use of the guidelines in the formulation of policy – arguably the use with the greatest potential impact.

Since their publication, the guidelines appear to have been used most frequently to conduct needs assessments. This use is consistent with the relatively recent publication of the guidelines and the fact that, in many countries, the systematization of trauma care is only just beginning. We identified only four countries – i.e. Ghana, India, Mexico and Viet Nam – in which use of the guidelines in a needs assessment had been followed-up with documentation of how the issues identified in the assessment had been addressed.^27^^,^^37^^,^^41^^,^^48^^,^^76^^,^^77^^,^^79^ Follow-up on other needs assessments is clearly an area for future research and advocacy.

Although WHO guidelines have been associated with weak stakeholder engagement,^119^ about one in every four implementation events that we traced involved endorsement of guidelines by at least one professional society. Ideally, with time, the main types of implementation events will shift away from data gathering and stakeholder endorsements towards more incorporation of the guidelines into educational curricula and health policy.

Over our study period, incorporation of the guidelines into educational interventions appeared to be a rare event – documented just 19 times overall and only once as a graduation requirement for resident physicians.^112^ The global dearth of formal trauma education for physicians was documented in 2009, in a survey of 774 final-year medical students in 77 countries; only 55% of the surveyed students reported they were comfortable providing basic trauma care.^120^ We recommend that the guidelines be incorporated into the mandatory degree requirements for medical professionals.

The WHO’s trauma care guidelines were developed specifically for guidance at health ministry level. The relative lack of the guidelines’ implementation at national policy level is therefore cause for concern. In the implementation of WHO guidelines, the interaction between researchers and health-care policy-makers has previously been identified as needing improvement.^121^ Our search revealed excellent examples of such interaction in Ghana, India and Mexico, where there had been national-level consensus meetings in which WHO trauma experts, trauma care professional societies and ministry of health representatives had collaborated to adapt the WHO trauma care guidelines to local circumstances.^27^ In addition to increased researcher and policy-maker interaction, the more intentional distribution of guidelines among policy-makers is a ready area for improvement. The findings of this systematic review indicate that the guidelines are most readily accessible in clinical journals or other types of information source that are probably accessed primarily by clinicians, not policy-makers.

In considering how to improve implementation of the trauma care guidelines, an article commissioned by WHO to address dissemination and implementation strategies might prove useful. This article states that WHO did not have a general, unified strategy for the dissemination and implementation of guidelines and that there was considerable room for improvement of the applicability, dissemination, implementation and timeliness of WHO guidelines.^121^ With regard to applicability, several of the information sources we included in our analysis commented specifically on the appropriateness of the guidelines for low- and middle-income countries.^122^^–^^125^ However, most of the implementation events we traced were reported in English-language information sources and none appeared to have been reported in Arabic – indicating a need for wider dissemination of guidelines among the countries, including most low- and middle-income countries, where English is not the predominant language. With regard to timing and timeliness, the dissemination of the guidelines we investigated coincided with an increasing awareness of the substantial contribution made by noncommunicable diseases in general – and injury in particular – to the global disease burden.^1^

This study has several limitations. Most importantly, given the chosen method, we cannot make any comment regarding the outcomes of any implementation. We can only state that the guidelines have been used in a certain way and cannot comment on the impact of that use. To assess the outcome of guideline implementation, further research – e.g. examination of process-of-care measures from sentinel sites where the guidelines have been adopted – is recommended. We made no effort to alleviate or evaluate concerns that the development of systems for trauma care might cause harm by diverting resources from other health systems. However, since injury has a disproportionate impact on people of working age, improving outcomes after injury is expected to have a substantial positive impact on a country’s overall resources.^1^ Furthermore, the trauma system development recommended in the WHO’s guidelines frequently entails a more efficient use of existing resources rather than an infusion of new ones. Finally, some improvements in trauma systems – e.g. in prehospital care, referral and patient transport networks and hospital staff training in patient triage and resuscitation – could be expected to benefit patients across a spectrum of acute-care pathologies, including obstetrics and cardiovascular and cerebrovascular diseases. Nonetheless, we acknowledge that, apart from one published report citing the beneficial effect of trauma system development on the outcomes of patients with ruptured aortic aneurysms,^126^ there is currently a lack of evidence that trauma system development improves health systems overall. Thus, thoughtful development of trauma systems should include the purposeful avoidance of: (i) duplication; (ii) distortions, such as the creation of a separate elite cohort of better-resourced health workers; (iii) disruptions, such as those caused by leaving posts vacant while health workers are trained; and (iv) distractions, such as specific reporting and other uncoordinated time-consuming tasks.^127^^,^^128^

Several of the authors in this study have an interest in reporting the implementation of the WHO’s guidelines. They attempted to minimize this potential source of bias by recruiting a co-author – who was not professionally involved with the topic or with the other authors or members of the advisory group – to review the implementation data independently.

An additional weakness of the study is the inclusion of only reports that were available electronically, via the Internet, or known to the 20 experts who were consulted. The use of the guidelines we investigated is likely to be considerably greater than the use we traced. Also, as we selected the experts who we would contact based on their frequent citation in the initial literature search, we failed to contact experts who have not published many articles. We decided to conduct a systematic review because we felt that remote surveys of stakeholders – which might, in theory, give a better balanced picture – were often associated with low response rates and inaccurate, anecdotal evidence. Although on-site interviews with stakeholders might allow more detailed investigation of trauma care guidelines in the future, they will require more labour and more resources than the systematic review we conducted.

Despite these limitations, this review adds substantially to the literature. It confirms that, as intended, WHO’s trauma care guidelines are being used in low- and middle-income countries across the globe, for needs assessments, education and policy development and with stakeholder endorsement. However, implementation of the guidelines has been documented in a minority of the WHO’s 194 Member States. Possible areas for high-yield and appropriate improvement in the implementation of the guidelines include increasing policy-makers’ awareness of the guidelines, incorporation of the guidelines into the formal education of most health-care providers, and systematic needs assessments based on the guidelines – to be followed by corrective action and ongoing monitoring.
